# The Responsibility of Silence

**Published:** 1909-11-20

**Authors:** 


*To the Editor of* The Hospital.

Sir,—Your recent articles on "The Re-election of Hospital Staffs" and "The Responsibility of Silence" show considerable ability in the construction of a *prima facie* case against the present over-adequate security of tenure of hospital medical appointments. Naturally enough, in the small space at disposal, the confuting of possible objections came last of all; perhaps it was performed rather too summarily. I refer especially to the contention that valuable work from hospital physicians or surgeons would never meet with misunderstanding prejudice on the part of their colleagues. Now we know well enough from history that medicine can furnish plenty of examples of what has been well called "the *via dolorosa* of the pioneer." The relations of John Hunter, of Sir James Paget, and many others, with their colleagues were at times those of much friction; and although, as your writer urges, ideas are more liberal now than then, yet still, human nature remains the same. The truth is, unfortunately, that a display of exceptional energy in any direction is bound to raise up detractors. In such cases as have been noticed, posterity, guided by knowledge of the ultimate event, with one voice condemns such detractors. But it is impossible for contemporary opinion to be so large-minded and discriminating; contemporaries have to put up with a good deal of which posterity never hears; and they can only guess at the future. In a scientific pursuit such as medicine the exceptional man is not always a pleasant sort of person. Swift's description of Lord Wharton was that he "never yet considered whether any proposition were true or false, but whether it were convenient for the present minute or company to affirm or deny it." Now our exceptional man is generally the very opposite of this; and although there is no doubt as to which of the two attitudes is the fine and worthy one, yet it is equally certain, too, which will be rewarded with immediate prosperity. Moreover, the courage necessary to the making of any advance often runs into extremes. People ahead of their time can be very blunt and very uncompromising—very "impossible." Let me relate an instance in a small way.

A member of a certain hospital staff was a fine surgeon, and, what is much rarer, he had a spark or two of the true fire of the investigator. After hard preliminary work, in a certain line, he made an advance, which, though much smaller than he himself thought it to be, was yet undeniable. But, using the hospital as a base, he bombarded the whole of the profession, home and colonial, with reprints of his papers; and some of them were returned inscribed with regrettably frank comments. Forgetting the maxim that the better the surgical workman the fewer his tools, he filled instrument-makers' catalogues with his modifications of simple implements. Worst of all, he never publicly disclaimed connection with accounts of his work in lay newspapers cuttings from which used to be brought to the hospital by patients. If at this time his name had come up for re-election, quite certainly there would have been some black balls in evidence, and possibly they might have predominated. Yet the general opinion was that he did more and, on the whole, better work than any of his then colleagues. I hope I have not at too great length indicated an obstacle for practical reformers to surmount.

Yours etc,

October 29. Observer.

